# Self-Reported Moderate-to-Vigorous Physical Activity: Its Association with Health-Related Quality of Life in a Large Cohort of People with Chronic Diseases

**DOI:** 10.3390/healthcare11233057

**Published:** 2023-11-28

**Authors:** Hosam Alzahrani, Najlaa Alotaibi, Adel Alshahrani, Khalid M. Alkhathami, Yasir S. Alshehri, Msaad Alzhrani, Fahad H. Alshehri, Rania Almeheyawi, Ibrahim Saeed Aljulaymi, Muhsen Alsufiany, Kabir P. Sadarangani, Hatem H. Allam, Barbara Barcaccia

**Affiliations:** 1 Department of Physical Therapy, College of Applied Medical Sciences, Taif University, Taif 21944, Saudi Arabia; 2 Department of Medical Rehabilitation Sciences-Physiotherapy Program, College of Applied Medical Sciences, Najran University, Najran 55461, Saudi Arabia; 3 Department of Health Rehabilitation, College of Applied Medical Sciences at Shaqra, Shaqra University, Shaqra 11961, Saudi Arabia; 4 Department of Physical Therapy, College of Medical Rehabilitation Sciences, Taibah University, Madinah 42353, Saudi Arabia; 5 Department of Physical Therapy and Health Rehabilitation, College of Applied Medical Sciences, Majmaah University, Majmaah 11952, Saudi Arabia; 6 Faculty of Health and Dentistry, School of Kinesiology, Universidad Diego Portales, Santiago 9170022, Chile; 7 Department of Kinesiology, Universidad Autónoma de Chile, Santiago 7500912, Chile; 8 Department of Psychology, Sapienza University of Rome, Via dei Marsi 78, 00185 Rome, Italy; 9 Associazione di Psicologia Cognitiva APC, Scuola di Psicoterapia Cognitiva srl SPC, Viale Castro Pretorio 116, 00185 Rome, Italy

**Keywords:** exercise, physical activity, chronic disease, cardiovascular disease, cancer, COPD, diabetes, health-related quality of life, SF-36, health survey

## Abstract

The aim of this study was to investigate the dose–response relationship between physical activity and health-related quality of life (HRQoL) in a large population-based sample of people with chronic disease. We analysed the data of 29,271 adults (15,315 women) who were diagnosed with chronic diseases and participated in the Welsh Health Survey (Wales, UK; data collection 2011–2015). Participants were classified, based on their weekly minutes of moderate-to-vigorous physical activity (MVPA), into four groups as follows: inactive (no MVPA), insufficiently active (<150 min/week), sufficiently active (≥150–<300), and very active (≥300). The main outcome was HRQoL measured via the Short-Form 36 Health Survey (SF-36). This study found a curvilinear association between MVPA and HRQoL and a dose–response relationship for the perception of general health and vitality domains. Compared to inactive participants, those who were very active had higher HRQoL scores (coefficient = 12.54; 95% confidence interval [CI] 11.39–13.70), followed by sufficiently active (coefficient = 11.70; 95% CI 10.91–12.49) and insufficiently active (coefficient = 9.83; 95% CI 9.15–10.51) participants. The fully adjusted regression model showed curvilinear associations between MVPA and the domains of SF-36. Future research should find ways to motivate people with chronic diseases to engage in physical activity. The evidence to support regular exercise in individuals with chronic diseases in all age groups is strong and compelling, and patients should be encouraged to regularly devote more time to physical activity in order to improve their health and well-being.

## 1. Introduction

In all age groups, physical inactivity is one of the most significant lifestyle factors associated with the development of chronic diseases and non-communicable diseases (NCDs) [1,2]. The impact of physical inactivity on the well-being of individuals is particularly interesting to investigate, considering its amenability to change; appropriate education and training can modify this unhealthy lifestyle factor.

In 2012, The Lancet published its first series on physical activity topics in order to increase awareness of the importance of physical activity in the prevention of NCDs [3]. Kohl and colleagues [3], in one of the articles in The Lancet series, noted that, considering the prevalence and detrimental effects of physical inactivity on health, this should be defined as a pandemic. As such, it has sweeping damaging health, social, and economic consequences. In actual fact, physical inactivity has become a pandemic in all age groups around the world, especially youngsters, leading to a significant increase in NCDs even in that age-range. Indeed, among other increases in NCDs, type 2 diabetes mellitus is overtaking type 1 diabetes as the predominant form of diabetes in children in Asia [4]. Recent estimates [5] forecast a decline in life expectancy in the next few decades due to the increasing growth of NCDs, such as cardiovascular disease, diabetes, stroke, cancer, and obesity.

Furthermore, worldwide data suggest a decline in life expectancy in the next few decades due to a rise in chronic diseases [5], which have recently become an emerging pandemic worldwide [6]. A previous report found that in 2016, more than 39 million (72.3%) of all global deaths were caused by chronic diseases [7]. The NCDs also represent the leading cause of premature deaths, accounting for approximately 60% of the mortality rate globally [8]. Chronic diseases result from a combination of behavioural, environmental, genetic, and physiological factors [6]. The main types of chronic diseases are cardiovascular diseases (e.g., stroke and heart attack), cancer, chronic respiratory diseases (e.g., asthma and obstructive pulmonary disease), and diabetes.

Chronic diseases are not only debilitating and burdensome for both patients and their family members, but they are also financially onerous [9]. Furthermore, they impose a considerable economic burden that is expected to become even bigger over the next few decades. Although the health and medicine community are concerned about the burden and expected increase in chronic diseases, policy-makers are less attentive to their effects on well-being, quality of life, development, and economic growth [10]. Nonetheless, the heavy burden of chronic diseases might be contained by devoting resources to their prevention, screening, and treatment. Such an investment would yield a considerable rate of return, as compellingly highlighted by Bloom and colleagues [10].

People with chronic diseases often report decreased functional health and emotional balance, fatigue, decreased exercise capacity, and mental distress [11]. Furthermore, research indicates that individuals with chronic diseases typically report decreased health-related quality of life (HRQoL), which might be in part a consequence of comorbidities and complications associated with their disease [12,13,14]. HRQoL is a multidimensional concept that incorporates physical, social, and psychological well-being [15,16].

Fortunately, numerous studies have shown that interventions could improve HRQoL in people with chronic disease [5,17,18]. One of the most promising interventions is physical activity, which has been found to be associated with good HRQoL and various physiological and psychological health benefits [15,19,20,21]. Exploring the role of physical activity is particularly relevant, since it represents a modifiable factor capable of improving HRQoL in individuals with chronic diseases.

Several studies have explored the relationship between physical activity and HRQoL in people with NCDs, showing positive associations between meeting recommended MVPA and HRQoL [22,23]. However, most of those studies included small sample sizes and did not adjust for covariates that may confound this association. Furthermore, to the best of our knowledge, none of the previous studies have investigated the dose–response relationship between physical activity and HRQoL. In order to gain health benefits, current physical activity guidelines consistently recommend engaging in at least 150 min per week of moderate-intensity physical activity, 75 min per week of vigorous-intensity physical activity, or an equivalent combination of both [19,20,24].

Previous research explored the dose–response relationship between physical activity and HRQoL in the general population [25], but there is still a need for a descriptive study including a large representative sample of people with chronic diseases. Such data will help in informing policy makers for developing effective physical activity interventions for improving HRQoL in people with chronic disease. The purpose of this study is to investigate the dose–response relationship between physical activity and HRQoL in a large population-based sample of people with chronic disease.

## 2. Methods

### 2.1. Participants and Procedure

We examined records from the Welsh Health Survey (WHS) (2011, 2012, 2013, 2014, and 2015), which includes 74,578 participants aged 16 years old or older. The WHS is a household/population-based survey that collects information annually regarding health status, illnesses, and health-related lifestyle on people living in Wales, United Kingdom. The WHS recruited participants continuously throughout the year using a multi-stage stratified random sample of addresses selected from the Postcode Address File. More details about the survey can be found elsewhere [26]. The WHS was approved by the NatCen Social Research Ethics Committee in London, United Kingdom. The data of this survey were anonymised and made available to bona fide researchers, and they can be accessed via the UK Data Archive (http://data-archive.ac.uk/). The data was accessed by the main author (HA) on 1 April 2021.

This study included participants who had been diagnosed with any of the four main type of NCDs, which include cardiovascular disease, type of cancer, COPD, and/or diabetes. The characteristics of all participants, with/without chronic diseases, are provided in the Supplementary Table S1. Approximately 34.9% of people without chronic diseases and 56.6% of people with chronic diseases are categorised as inactive. The average of HRQoL was higher in people without chronic diseases (80.03 ± 16.85) than those with chronic diseases (64.55 ± 23.91).

**Figure 1:**
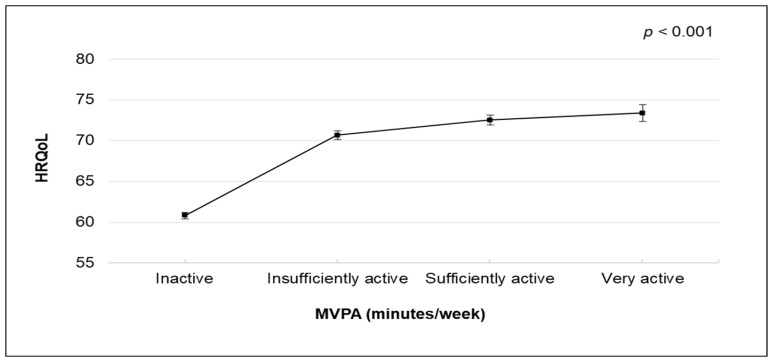
Multivariable-adjusted means and 95% CIs of HRQoL by MVPA volume (very active: reporting ≥300 min/week; sufficiently active: reporting ≥150–300 min/week; insufficiently active: reporting >0–150 min/week; inactive: not reporting any MVPA). The model was adjusted for sex, age, education, employment, BMI, smoking status, musculoskeletal conditions, mental illness, and light-intensity physical activity. Abbreviations: BMI, body mass index. HRQoL, health-related quality of life. MVPA, moderate-to-vigorous-intensity physical activity.

The characteristics of the included participants, categorised according to their physical activity level, are shown in Table 1. Of 74,578 participants included in the WHS cohorts, 29,271 (39.2%) had a diagnosis of chronic disease and were included in the analyses (Figure 1). Around 50.8% of the participants were 65 years old or older, 52.3% were females, 44.3% had never smoked, 56.5% had an education level lower than a tertiary degree, 69.5% were unemployed, 83.8% did not have mental illnesses, and 61.3% did not have musculoskeletal problems. In total, 56.6% of the sample was composed of inactive participants, and they were more likely to be older than 65 years old, female, and people who had never smoked.

### 2.2. Chronic Diseases

The data about chronic diseases in the survey were collected via two questions: (A) “Are you currently being treated for any of these?” and (B) “Have you ever been treated for any of these?”. Participants were included in the study if they were currently being treated or had ever been treated for any cardiovascular disease, type of cancer, COPD, and/or diabetes (i.e., those who answered “yes” to either question A or question B).

### 2.3. Physical Activity

Participants had been asked to report the number of days over the previous week in which they participated in physical activity or exercise of any intensity for a minimum of 30 min (or accumulated over several bouts lasting a minimum of 10 min performed on the same day). The intensity of the physical activity/exercise was classified based on the following measures: light-intensity exercise (e.g., walking, golfing, and simple homework such as vacuuming), moderate-intensity exercise (e.g., cycling, tennis, and playing with children at home), and vigorous-intensity exercise (e.g., running and playing basketball and volleyball). The questions section regarding physical activity used in the survey is presented in Supplementary Table S2.

Participants were classified into four groups according to their adherence to current physical activity guidelines for moderate- or vigorous-intensity activity or an equivalent combination of both types [19,20]. The equivalent combination was calculated using the following equation: [(vigorous-intensity activity × 2) + moderate-intensity activity]. This calculation method was utilised in previous research studies [27,28,29]. Subjects have been classified into various groups: inactive (not engaging in any MVPA), insufficiently active (reporting <150 min per week of MVPA), sufficiently active (reporting ≥150 and <300 min per week of MVPA), and highly active (reporting ≥300 min per week of MVPA).

### 2.4. Health-Related Quality of Life

The HRQoL was measured using the 36-item short form (SF-36) [22]. The SF-36 scale consists of eight domains: physical functioning (10 items), bodily pain (2 items), role limitations due to physical health problems (4 items), role limitations due to emotional problems (3 items), emotional health well-being (5 items), social functioning (2 items), vitality or energy/fatigue (4 items), and general health perceptions (5 items). The score for each domain ranged between 0 and 100. The overall HRQoL score was calculated by taking the average of the eight domains (range 0–100) [30]. The higher the score, the better the HRQoL. More information about SF-36 form is provided elsewhere [31,32].

### 2.5. Potential Confounding Variables

The confounding variables that may impact the HRQoL were identified based on the existing literature and included age, sex, body mass index (BMI), musculoskeletal disorders, mental illness, smoking, employment status, and education. The BMI was calculated from the self-reported height and weight, and participants were categorised as underweight [<18.5 kg/m^2^], normal weight [18.5–24.9 kg/m^2^], overweight [25.0–29.9 kg/m^2^], or obese [≥30.0 kg/m^2^].

### 2.6. Statistical Analyses

The primary analysis in this study was performed to evaluate the relationship between MVPA and HRQoL. The secondary analysis was conducted to evaluate the relationship between MVPA and the SF-36 subdomains. Generalised linear models were used in all analyses, along with multiple linear regression to detect linear trend *p* values. Different models were adjusted for the following factors: (1) age and sex; (2) BMI, education level, smoking status, employment, mental illness, musculoskeletal disorder, and light-intensity physical activity. The coefficients of the generalised linear model reveal the differences in mean HRQoL between the reference group (inactive) and each of the other MVPA groups. Secondary analyses were also conducted by measuring the association between MVPA and HRQoL, categorised by the main types of NCD, age groups, or sexes. The analyses were performed in the period April–May 2021 using the IBM SPSS statistics software (version 22; Armonk, NY, USA). The statistical test was considered significant when recording a *p* value < 0.05.

## 3. Results

### Physical Activity and Health-Related Quality of Life

The relationship between MVPA and HRQoL is shown in Table 2 (and Figure 1). The maximally adjusted linear regression model demonstrated a curvilinear relationship between MVPA and HRQoL (Figure 1). The difference in the overall HRQoL score by physical activity level was decreased, but the significant association persisted (Table 2). Compared to inactive participants, those who were very active had higher HRQoL scores (coefficient = 12.54; 95% confidence interval [CI] 11.39–13.70), followed by individuals who were sufficiently active (coefficient = 11.70; 95% CI 10.91–12.49) and insufficiently active (coefficient = 9.83; 95% CI 9.15–10.51). When we conducted secondary analyses by measuring the association between MVPA and HRQoL, categorised by the main types of NCD, ages groups, or sexes, we found a similar trend to the main analysis (Supplementary Tables S3–S5).

**Figure 2:**
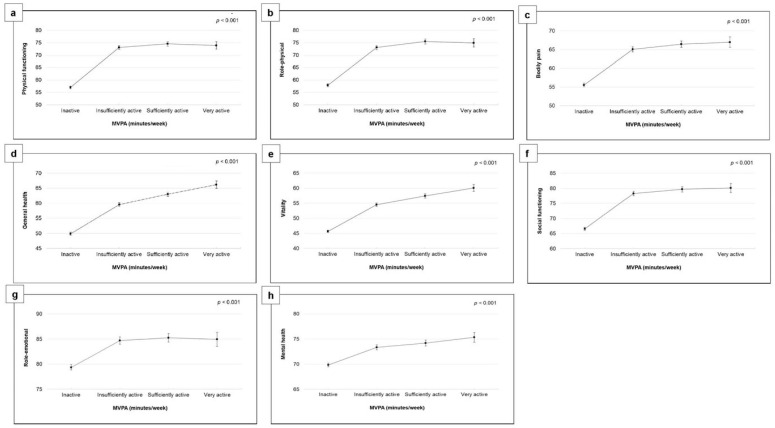
Multivariable-adjusted means and 95% CIs of SF-36 domains by MVPA volume (Very active: reporting ≥300 min/week; Sufficiently active: reporting ≥150–300 min/week; Insufficiently active: reporting >0–150 min/week; Inactive: not reporting any MVPA). The model was adjusted for sex, age, education, employment, BMI, smoking status, musculoskeletal conditions, mental illness and light-intensity physical activity. Abbreviations: BMI, body mass index; HRQoL, health-related quality of life; MVPA, moderate-to-vigorous-intensity physical activity. SF-36 domains: (**a**) Physical functioning, (**b**) Role-physical, (**c**) Bodily pain, (**d**) General health, (**e**) Vitality, (**f**) Social functioning, (**g**) Role-emotional, (**h**) Mental health.

The results revealed consistent direct relationships between MVPA and all SF-36 domains; however, these associations were weakened after controlling for all potential confounding factors (Table 2). Furthermore, the fully adjusted linear regression (model 2) showed curvilinear associations between MVPA and all the domains of SF-36 (Figure 2). Specifically, in comparison to the inactive group, being “very active” was associated with a higher score on the role-physical (coefficient = 17.04; 95%CI 15.21–18.86), physical functioning (coefficient = 16.86; 95%CI 15.21–18.50), and general health (coefficient = 16.29; 95%CI 14.96–17.62) measures. Moreover, those who engaged in any level of physical activity achieved a higher score on the SF-36 domains compared to those who did not engage in any level of activity (inactive).

## 4. Discussion

Our study investigated the association between MVPA and HRQoL in individuals with chronic diseases. The results clearly demonstrated a direct and curvilinear association between MVPA and HRQoL, and even the insufficiently active participants had better HRQoLs compared to inactive participants. The results showed that higher levels of MVPA were associated with higher scores on each SF-36 subdomain. These results are consistent with the results of previous studies, which had found that engaging in higher levels of physical activity is associated with better HRQoL among individuals with chronic disease [22,23]. In particular, there was a dose–response relationship for general health perception and vitality (energy/fatigue) domains. This result is in line with those already found in a previous study of the associations between physical activity and HRQoL in the general population [25]. However, the author found that the dose–response relationship considered all the SF-36 domains, e.g., physical pain and role limitations due to physical health. These variables, in individuals with chronic diseases, are heavily affected by other dimensions in relation to their physical illness. Physical activity is not only important for their medical condition, but it is also important to improve two relevant facets of HRQoL, general health perception, and vitality, with the dose–response relationship representing a fundamental aspect.

Our findings confirm the results of previous studies of the role of physical activity in chronic disease by examining the association between different levels of physical activity and HRQoL. Although a previous prospective study of adults with type 2 diabetes showed that those who met the MVPA recommendations had better quality of life [23], our findings demonstrate that even little physical activity is better than none, and it is capable of yielding health benefits. However, our results highlight the importance of achieving the recommended levels of MVPA for a better HRQoL.

In our study, 50.8% of the sample was composed of people aged 65 years old or older, which was to be expected, as it is the group with the highest prevalence of chronic diseases, specifically those related to low levels of physical activity [33]. Zhao and colleagues [34] demonstrated that the recommended levels of exercise should be met in order to achieve greater survival rates. The authors also brought to light the scarcity of education programs including information on the health benefits of physical activity and the paucity of adequate affordable facilities [32].

Furthermore, it has to be considered that while, in the past, NCDs were considered typical of the elderly population, they are now becoming more and more prevalent in younger adults due to lifestyle changes, primarily physical inactivity [35]. These lifestyle modifications are accountable for the earlier onset of chronic diseases [9]. While the association between aging and NCDs is well established [36], the rise in younger age groups is troubling. A possible explanation of the increase in the prevalence of chronic diseases in younger age ranges is related to unhealthy habits developed in adolescence. Indeed, adolescents typically engage in low levels of physical activity, sedentary behaviour, unhealthy diet, and smoking [9,37].

Moreover, the prevalence of chronic diseases, such as type 2 diabetes, and risk factors for the development of chronic diseases, such as obesity and hypertension, are increasing among youngsters [37]. Future studies could also further investigate the explored variables in younger age groups, as well as the roles of other variables mediating the relationship between chronic disease and physical activity, such as the presence of social support. In a study of a sample of adults with chronic obstructive pulmonary disease, living with others was associated with higher levels of physical activity, indicating that social environment could be a potential asset to enhance patients’ engagement in healthy self-care habits [38].

The aim of the current physical activity recommendations is to promote exercise among people across all age groups. Since the first version of the recommendations was published [19], knowledge of the fundamental role of exercise in improving health has been spreading, and many other countries have issued their own guidelines [9]. Nevertheless, the level of physical activity has not increased in the following years; in fact, it has declined [39]. Apparently, the knowledge stemming from numerous robust studies of the benefits of physical activity for health has not yet been translated into action [40]. In this respect, at the public level, health campaigns should be launched to raise awareness of the importance of exercise.

The strengths of our study were its large representative sample size (a country-wide and population-based sample was used) and the assessment of HRQoL using SF-36, one of the most utilised instruments of measure worldwide with excellent psychometric properties. However, in this study, there are a few limitations that cannot be ignored. Firstly, due to the cross-sectional study design, a causal relationship between variables cannot be established. Future experimental studies could explore the direction of this relationship. Another possible explanation could be the circularity of this relationship: instead of a linear causal link, physical activity and vitality/general health perception might affect one another in a virtuous circle mechanism. Secondly, our findings were based on self-reported measures, which might over- or under-estimate physical activity or HRQoL. Thirdly, there was a lack of information on specifical types of physical activity that participants used to undertake before the onset of their chronic disease. Fourthly, the physical activity data were collected with a specific questionnaire that was not validated previously; therefore, future studies are recommended to use a lifetime physical activity questionnaire, which might have more accurate data and note an important relationship between physical activity and HRQoL, especially in people with chronic diseases. Fifthly, some information regarding the chronic disease, such as the severity of disease and the medications used by participants, were not available, which might have had an impact on HRQoL, consequently confounding the association between physical activity and HRQoL.

Despite these limitations, our results clearly demonstrate a positive association between MVPA and HRQoL. Even the participants who were insufficiently active had a better HRQoL compared to those who were inactive. In particular, higher levels of physical activity were associated with higher scores on the eight domains of the SF-36. Our results confirm the findings of previous research showing that higher levels of physical activity are associated with better HRQoL in individuals with chronic disease [22,23]. They add to the accumulating evidence for the importance of physical activity in people with NCDs, showing a dose–response relationship for general health perception and vitality. In particular, we examined the association between different levels of physical activity and HRQoL and found that even little physical activity is better than none, and it is capable of yielding health and well-being benefits.

In summary, our findings offer a wide spectrum of data related to health, functioning, and psychological well-being in individuals with NCDs, opening up new opportunities for further research in this field. In our study, individuals with chronic diseases had significantly lower HRQoLs compared to individuals without chronic diseases. The large majority of participants with chronic diseases were either inactive or insufficiently active. The results of our study add to previous research on the benefits of physical activity for maintaining high levels of well-being and quality of life, even in the face of physical health burdens.

Future research should find ways to motivate people with chronic diseases to engage in physical activity. Public health and policy interventions should create feasible, acceptable, and sustainable physical-activity-promoting environments. The evidence to support regular exercise in all age groups is strong and compelling, and everyone, particularly those affected by chronic diseases, should be encouraged to regularly devote more time to physical activity in order to improve their health and well-being [41].

## 5. Conclusions

The results showed a direct and curvilinear relationship between MVPA and HRQoL, with even the insufficient active participants reporting higher HRQoLs than those who were inactive. Higher levels of MVPA were also correlated with better scores on the eight SF-36 domains.

## Figures and Tables

**Table 1:** Baseline characteristics of participants recruited from the Welsh Health Survey (2011–2015).

		MVPA (min/week)				
Characteristics	All Participants (n = 29,271)	Inactive (n = 16,569)	Insufficiently Active (n = 6414)	Sufficiently Active (n = 4703)	Very Active (n = 1585)	*p* Value *
	N (%)	%	%	%	%	
Age (years)						<0.001
16–24	1014 (3.5)	2.0	4.0	5.1	12.0	
25–34	1180 (4.0)	2.6	5.4	5.2	9.5	
35–44	2053 (7.0)	5.3	7.8	9.8	13.2	
45–54	3815 (13.0)	11.2	13.6	16.4	20.3	
55–64	6346 (21.7)	20.1	23.2	25.3	21.5	
≥65	14,863 (50.8)	58.8	46.0	38.2	23.6	
Gender						<0.001
Male	13,956 (47.7)	45.6	43.3	54.6	66.5	
Female	15,315 (52.3)	54.4	56.7	45.4	33.5	
Body mass index						<0.001
Underweight (<18.5)	428 (1.6)	2.0	0.9	1.1	1.1	
Normal (18.5–<25)	8149 (30.0)	28.5	30.4	32.5	36.3	
Overweight (25–<30)	10,387 (38.2)	36.0	40.9	41.0	42.1	
Obese (≥30)	8193 (30.2)	33.4	27.9	25.4	20.5	
Smoking status						<0.001
Never smoked	12,758 (44.3)	41.7	48.1	46.1	50.2	
Ex-smoker	11,223 (38.9)	40.5	37.2	37.4	34.5	
Current smoker	4842 (16.8)	17.9	14.7	16.5	15.3	
Highest level of qualification						<0.001
Degree or above	3173 (11.9)	8.7	16.4	14.4	18.5	
Other qualification	15,010 (56.5)	51.1	62.0	63.6	66.6	
No qualification	8389 (31.6)	40.3	21.6	22.0	14.9	
Employment status						<0.001
Currently employed	8457 (30.5)	20.3	36.2	46.4	61.9	
Not employed	19,289 (69.5)	79.7	63.8	53.6	38.1	
Prevalent mental illness						<0.001
No	22,599 (83.8)	79.2	88.3	90.4	91.7	
Yes	4377 (16.2)	20.8	11.7	9.6	8.3	
Prevalent musculoskeletal problems						0.004
No	16,912 (61.3)	61.3	59.6	62.4	63.6	
Yes	10,695 (38.7)	38.7	40.4	37.6	36.4	
Cardiovascular disease						<0.001
No	9512 (32.5)	27.4%	35.8%	39.5%	51.7%	
Yes	19,759 (67.5)	72.6%	64.2%	60.5%	48.3%	
Cancer						<0.001
No	22,506 (83.5)	82.1%	84.8%	85.7%	86.5%	
Yes	4437 (16.5)	17.9%	15.2%	14.3%	13.5%	
COPD						<0.001
No	19,164 (65.5)	65.3%	67.8%	66.0%	56.7%	
Yes	10,107 (34.5)	34.7%	32.2%	34.0%	43.3%	
Diabetes						<0.001
No	23,316 (80.3)	77.4%	83.1%	84.4%	87.3%	
Yes	5709 (19.7)	22.6%	16.9%	15.6%	12.7%	

Abbreviations: MVPA: moderate-to-vigorous-intensity physical activity. Inactive: not reporting any MVPA. Insufficiently active: reporting >0–150 min/week. Sufficiently active: reporting ≥150–300 min/week. Very active: reporting ≥300 min/week. * Chi-squared tests for group differences.

**Table 2:** Multivariable-adjusted associations between MVPA and the HRQoL and SF-36 domains in people with chronic disease.

	Model 1 ^a^	Model 2 ^a^
	Coefficient (95% CI)	Coefficient (95% CI)
HRQoL ^b^		
Inactive	Referent	Referent
Insufficiently active	17.22 (16.56–17.88)	9.83 (9.15–10.51)
Sufficiently active	21.28 (20.54–22.02)	11.70 (10.91–12.49)
Very active	23.99 (22.82–25.16)	12.54 (11.39–13.70)
Trend *p* value	<0.001	<0.001
SF-36 domains		
Physical functioning ^b^		
Inactive	Referent	Referent
Insufficiently active	25.84 (24.96–26.71)	16.05 (15.09–17.01)
Sufficiently active	30.82 (29.84–31.81)	17.51 (16.39–18.62)
Very active	32.71 (31.12–34.29)	16.86 (15.21–18.50)
Trend *p* value	<0.001	<0.001
Role-physical ^b^		
Inactive	Referent	Referent
Insufficiently active	24.57 (23.61–25.54)	15.15 (14.08–16.22)
Sufficiently active	30.33 (29.24–31.42)	17.57 (16.32–18.82)
Very active	32.45 (30.71–34.18)	17.04 (15.21–18.86)
Trend *p* value	<0.001	<0.001
Role-emotional ^b^		
Inactive	Referent	Referent
Insufficiently active	15.02 (14.11–15.93)	5.34 (4.42–6.26)
Sufficiently active	18.22 (17.20–19.24)	5.89 (4.82–6.97)
Very active	19.83 (18.20–21.46)	5.59 (4.01–7.16)
Trend *p* value	<0.001	<0.001
Vitality ^b^		
Inactive	Referent	Referent
Insufficiently active	14.08 (13.43–14.72)	8.84 (8.10–9.58)
Sufficiently active	18.62 (17.89–19.35)	11.76 (10.90–12.62)
Very active	22.81 (21.64–23.99)	14.42 (13.15–15.69)
Trend *p* value	<0.001	<0.001
Mental health ^b^		
Inactive	Referent	Referent
Insufficiently active	9.10 (8.50–9.69)	3.50 (2.87–4.13)
Sufficiently active	11.10 (10.43–11.77)	4.36 (3.62–5.10)
Very active	13.73 (12.65–14.81)	5.49 (4.41–6.57)
Trend *p* value	<0.001	<0.001
Social functioning ^b^		
Inactive	Referent	Referent
Insufficiently active	21.51 (20.63–22.38)	11.70 (10.74–12.65)
Sufficiently active	25.79 (24.80–26.78)	13.08 (11.96–14.20)
Very active	28.53 (26.94–30.12)	13.56 (11.91–15.20)
Trend *p* value	<0.001	<0.001
Bodily pain ^b^		
Inactive	Referent	Referent
Insufficiently active	15.95 (15.14–16.77)	9.48 (8.56–10.39)
Sufficiently active	19.68 (18.76–20.60)	10.87 (9.80–11.93)
Very active	22.51 (21.03–23.99)	11.40 (9.82–12.97)
Trend *p* value	<0.001	<0.001
General health ^b^		
Inactive	Referent	Referent
Insufficiently active	14.54 (14.86–16.23)	9.70 (8.92–10.47)
Sufficiently active	20.56 (19.78–21.33)	13.13 (12.22–14.03)
Very active	25.45 (24.21–26.69)	16.29 (14.96–17.62)
Trend *p* value	<0.001	<0.001

Abbreviations: BMI, body mass index; CI, confidence interval. HRQoL, health-related quality of life. MVPA, moderate-to-vigorous-intensity physical activity. Scale range for HRQoL and SF-36 subdomains: 0–100; higher scores indicate a better health status. Inactive: not reporting any MVPA. Insufficiently active: reporting >0–150 min/week. Sufficiently active: reporting ≥150–300 min/week. Very active: reporting ≥300 min/week. ^a^ Model 1: adjusted for sex and age. Model 2: further adjustment for BMI, smoking status, education, employment, musculoskeletal conditions, mental illness, and light-intensity physical activity. ^b^ Generalised linear model coefficients; coefficients indicate the mean differences (in HRQoL and SF-36 domains) between the reference category (inactive) and each of the other MVPA groups, e.g., a value of three indicates that a specific category had a mean score that is three units higher than that of the referent group.

## Data Availability

The data were anonymised and made available to bona fide researchers via the UK Data Archive (http://data-archive.ac.uk/). The data was accessed by the main author (HA) on 1 April 2021.

## Supplementary Materials

The following supporting information can be downloaded via this link: https://www.mdpi.com/article/10.3390/healthcare11233057/s1, Table S1: The characteristics of all participants, with/without chronic diseases.; Table S2: Physical activity questions used in the Welsh Health Survey. Table S3: Multivariable adjusted associations between MVPA and HRQoL in people with cardiovascular diseases, cancer, COPD, and diabetes. Table S4: Multivariable-adjusted associations between MVPA and HRQoL in people with chronic diseases, categorised by ages group. Table S5: Multivariable-adjusted associations between MVPA and HRQoL in people with chronic diseases, categorised by sex.
