# Supplementary material for: Self-Reported Moderate-to-Vigorous Physical Activity: Its Association with Health-Related Quality of Life in a Large Cohort of People with Chronic Diseases
Source: Healthcare (Basel). 2023 Nov 28;11(23):3057. doi: 10.3390/healthcare11233057 (PMC10706350; doi:10.3390/healthcare11233057)
Supplement: Supplementary file 1 [file healthcare-11-03057-s001.zip › healthcare-2633695-supplementary.pdf]

Supplementary Materials

Table S1. The characteristics of all participants, with/without chronic diseases.

	All participants	People without chronic diseases	People with chronic diseases	p value
**Physical activity level, N (%)**				< 0.001
Inactive	32398 (43.4)	15829 (34.9)	16569 (56.6)	
Insufficiently active	18486 (24.8)	12072 (26.6)	6414 (21.9)	
Sufficiently active	15927 (21.4)	11224 (24.8)	4703 (16.1)	
Very active	7767 (10.4)	6182 (13.6)	1585 (5.4)	
**HRQoL, mean±SD**	74.23±21.16	80.03±16.85	64.55±23.91	< 0.001

Abbreviations: HRQoL, health-related quality of life; MVPA, moderate- to vigorous-intensity physical activity; SD, standard deviation.

Scale range for HRQoL: 0-100, higher scores indicative of better status or health.

Inactive: not reporting any MVPA; Insufficiently activ e: reporting > 0 – 150 min/week; Sufficiently active: reporting ≥ 150 – 300 min/week; Very active: reporting ≥ 300 min/week.

Table S2. Physical activity questions used in the Welsh Health Survey.

EXERCISE

43a During the **7 days ending yesterday**, on which days did you do **LIGHT** exercise or physical activity **for at least 30 minutes**?
Blocks of activity lasting at least 10 minutes, which were done on the same day, can be counted towards the full 30 minutes.
Include physical activity which is part of your job

exltnum...exltmon, exlttue, exltwed, exltthu, exltfri, exltsat, exltsun

Tick all days that apply

	Mon	Tue	Wed	Thu	Fri	Sat	Sun	
**Light exercise / activity** ***For example*** Housework (eg hoovering, dusting), walking at an average pace, golf, light gardening (eg weeding)	☐ 01	☐ 02	☐ 03	☐ 04	☐ 05	☐ 06	☐ 07	2451-64
If no light exercise in the **last 7 days**, please tick	☐ 08 exltno							

43b During the **7 days ending yesterday**, on which days did you do **MODERATE** exercise or physical activity **for at least 30 minutes**?
Blocks of activity lasting at least 10 minutes, which were done on the same day, can be counted towards the full 30 minutes.
Include physical activity which is part of your job

exmodnum...exmodmon, exmodtue, exmodwed, exmodthu, exmodfri, exmodsat, exmodsun

Tick all days that apply

	Mon	Tue	Wed	Thu	Fri	Sat	Sun	
**Moderate exercise / activity** ***For example*** Heavy housework (eg spring cleaning, walking with heavy shopping), fast walking, dancing, gentle swimming, heavy gardening (eg digging)	☐ 01	☐ 02	☐ 03	☐ 04	☐ 05	☐ 06	☐ 07	2465-78
If no moderate exercise in the **last 7 days**, please tick	☐ 08 exmodno							

43c During the **7 days ending yesterday**, on which days did you do **VIGOROUS** exercise or physical activity **for at least 30 minutes**?
Blocks of activity lasting at least 10 minutes, which were done on the same day, can be counted towards the full 30 minutes.
Include physical activity which is part of your job

exvignum ...exvigmon, exvigtue, exvigwed, exvigthu, exvigfri, exvigsat, exvigsun

Tick all days that apply

	Mon	Tue	Wed	Thu	Fri	Sat	Sun	
**Vigorous exercise / activity** ***For example*** Running, jogging, squash, swimming lengths, aerobics, fast cycling, football	☐ 01	☐ 02	☐ 03	☐ 04	☐ 05	☐ 06	☐ 07	2479-92
If no vigorous exercise in the **last 7 days**, please tick	☐ 08 exvigno							

Table S3. Multivariable adjusted associations between MVPA and HRQoL in people with cardiovascular, cancer, COPD and diabetes diseases.

	**HRQoL** [a b]
	Coefficient (95% CI)
**People with cardiovascular diseases**	
Inactive	Referent
Insufficiently active	10.35 (9.51, 11.20)
Sufficiently active	12.12 (11.12, 13.13)
Very active	12.45 (10.82, 14.09)
Trend p value	< 0.001
**People with cancer**	
Inactive	Referent
Insufficiently active	12.60 (10.77, 14.43)
Sufficiently active	15.09 (12.94, 17.23)
Very active	15.39 (12.13, 18.65)
Trend p value	< 0.001
**People with COPD**	
Inactive	Referent
Insufficiently active	9.30 (8.12, 10.48)
Sufficiently active	11.39 (10.04, 12.74)
Very active	12.79 (11.01, 14.57)
Trend p value	< 0.001
**People with diabetes**	
Inactive	Referent
Insufficiently active	11.68 (10.01, 13.35)
Sufficiently active	13.34 (11.30, 15.38)
Very active	12.94 (9.65, 16.24)
Trend p value	< 0.001

Abbreviations: CI, confidence interval; COPD, chronic obstructive pulmonary disease; HRQoL, health-related quality of life; MVPA, moderate- to vigorous-intensity physical activity.

Scale range for HRQoL and each SF-36 domain: 0-100, higher scores indicative of better status or health.

Inactive: not reporting any MVPA; Insufficiently activ e: reporting > 0 – 150 min/week; Sufficiently active: reporting ≥ 150 – 300 min/week; Very active: reporting ≥ 300 min/week.

[a] adjusted for body mass index, education, employment, smoking status, mental illness, musculoskeletal conditions, and light-intensity physical activity.
[b] Generalized linear model coefficients; coefficients indicate mean differences (in HRQoL and SF-36 domains) between the reference category (Inactive) and each of the other MVPA groups, e.g., a value of three indicates that a specific category had a mean score that is three units higher than the referent group.

Table S4. Multivariable adjusted associations between MVPA and HRQoL in people with chronic diseases, categorised by ages groups.

	**HRQoL** [a b]
	Coefficient (95% CI)
**Ages, years**	
**16-39**	
Inactive	Referent
Insufficiently active	4.72 (2.92, 6.52)
Sufficiently active	7.42 (5.46, 9.38)
Very active	9.09 (6.82, 11.36)
Trend p value	< 0.001
**40-59**	
Inactive	Referent
Insufficiently active	9.28 (8.10, 10.47)
Sufficiently active	11.10 (9.78, 12.42)
Very active	13.08 (11.34, 14.82)
Trend p value	< 0.001
**≥ 60**	
Inactive	Referent
Insufficiently active	10.53 (9.63, 11.43)
Sufficiently active	12.95 (11.86, 14.05)
Very active	13.52 (11.55, 15.50)
Trend p value	< 0.001

Abbreviations: CI, confidence interval; COPD, chronic obstructive pulmonary disease; HRQoL, health-related quality of life; MVPA, moderate- to vigorous-intensity physical activity.

Scale range for HRQoL and each SF-36 domain: 0-100, higher scores indicative of better status or health.

Inactive: not reporting any MVPA; Insufficiently activ e: reporting > 0 – 150 min/week; Sufficiently active: reporting ≥ 150 – 300 min/week; Very active: reporting ≥ 300 min/week.

[a] adjusted for body mass index, education, employment, smoking status, mental illness, musculoskeletal conditions, and light-intensity physical activity.
[b] Generalized linear model coefficients; coefficients indicate mean differences (in HRQoL and SF-36 domains) between the reference category (Inactive) and each of the other MVPA groups, e.g., a value of three indicates that a specific category had a mean score that is three units higher than the referent group.

Table S5. Multivariable adjusted associations between MVPA and HRQoL in people with chronic diseases, categorised by sexes.

	**HRQoL** [a b]
	Coefficient (95% CI)
**Sexes**	
**Male**	
Inactive	Referent
Insufficiently active	9.18 (8.21, 10.14)
Sufficiently active	11.20 (10.13, 12.27)
Very active	12.28 (10.84, 13.73)
Trend p value	< 0.001
**Female**	
Inactive	Referent
Insufficiently active	10.35 (9.397, 11.303)
Sufficiently active	12.28 (11.11, 13.45)
Very active	12.93 (10.98, 14.88)
Trend p value	< 0.001

Abbreviations: CI, confidence interval; COPD, chronic obstructive pulmonary disease; HRQoL, health-related quality of life; MVPA, moderate- to vigorous-intensity physical activity.

Scale range for HRQoL and each SF-36 domain: 0-100, higher scores indicative of better status or health.

Inactive: not reporting any MVPA; Insufficiently activ e: reporting > 0 – 150 min/week; Sufficiently active: reporting ≥ 150 – 300 min/week; Very active: reporting ≥ 300 min/week.

[a] adjusted for body mass index, education, employment, smoking status, mental illness, musculoskeletal conditions, and light-intensity physical activity.
[b] Generalized linear model coefficients; coefficients indicate mean differences (in HRQoL and SF-36 domains) between the reference category (Inactive) and each of the other MVPA groups, e.g., a value of three indicates that a specific category had a mean score that is three units higher than the referent group.
